# Narrative Divergence and Disinformation: An Entropic Model for Assessing the Informative Utility of Public Information Sources

**DOI:** 10.3390/e28020183

**Published:** 2026-02-06

**Authors:** José Ignacio Peláez, Gustavo Fabian Vaccaro, Felix Infante León

**Affiliations:** 1 Center for Applied Social Research (CISA), University of Málaga, 29010 Málaga, Spain; fabianvaccaro@uma.es (G.F.V.); finfantel@uma.es (F.I.L.); 2 Department of Languages and Computer Science, University of Málaga, 29071 Málaga, Spain; 3 Biomedical Research Institute of Málaga (IBIMA), University of Málaga, 29071 Málaga, Spain

**Keywords:** disinformation modeling, narrative divergence, informational entropy, semantic distance, informative utility, epistemic coherence, natural language processing (NLP)

## Abstract

In today’s information ecosystem, disinformation threatens civic autonomy and the stability of public discourse. Beyond the intentional spread of false information, it often appears as narrative divergence among sources interpreting shared events, generating fragmentation and measurable losses in structural coherence. This study examines disinformation within an entropic structural framework, defining it as narrative disorder and epistemic incoherence in information systems. The approach moves beyond fact-checking by treating narrative structure and informational order as quantifiable attributes of public communication. We present the QVP-RI (Relational Information Valuation) operator, a computational model that quantifies narrative divergence through informational entropy and normalized structural divergence, without issuing truth assessments. Implemented through state-of-the-art NLP pipelines and entropic analysis, the operator maps narrative structure and epistemic order across plural media environments. Unlike accuracy-driven approaches, it evaluates narrative coherence and informational utility (IU) as complementary indicators of epistemic value. Experimental validation with 500 participants confirms the robustness of the structural–entropic model and identifies high divergence regions, revealing communication vulnerabilities and showing how narrative disorder enables disinformation dynamics. The QVP-RI operator thus offers a computationally grounded tool for analyzing disinformation as narrative divergence and for strengthening epistemic order in open information systems.

## 1. Introduction

Disinformation constitutes one of the significant contemporary challenges for democratic societies, as it simultaneously undermines citizen autonomy, the quality of public debate, and social cohesion. In today’s digital environment, its impact extends far beyond the mere dissemination of falsehoods. It is manifested through the spread of structurally divergent narratives that compete for the interpretation of events and the framing of shared reality. This process ultimately undermines trust in institutions and accentuates polarization, to the point of fracturing public discourse into narratives that are difficult to reconcile, whether because of hidden interests, explicit editorial lines, cultural biases, informational production dynamics, or implicit ideological frameworks [1,2,3,4,5,6].

Although intensified by the acceleration of digital platforms, this dynamic is not new. A well-documented historical example is the “Black Legend”, a campaign of distorted narratives promoted by political and economic adversaries against the Spanish Monarchy in the sixteenth century. Its purpose was to erode Spain’s international legitimacy through the construction of a persistent and emotionally charged narrative [7]. Viewed retrospectively, this case anticipates, within a historical frame, the exact mechanisms of structural disinformation that now operate within contemporary digital media ecosystems.

Recent reports, such as the Global Risks Report of the World Economic Forum [8], rank disinformation among the ten most significant global risks, warning of its negative impact on social cohesion, critical thinking, and collective decision-making processes. Complementarily, a bibliometric review [9], encompassing over 5600 papers published between 2002 and 2021, confirms that disinformation has become a priority field in international academic research, with particular emphasis on the mechanisms of propagation across social networks, the role of users in content dissemination, and the use of algorithmic, data-mining, and big data techniques for analyzing the infodemic. This landscape reinforces the view that disinformation must be understood as a complex, structural, and narrative phenomenon, in which the struggle for meaning is as consequential as the fabrication of facts.

In response to this threat, two main approaches have emerged. On the one hand, normative initiatives seek to establish centralized mechanisms of truth verification, generally promoted by public institutions or entities linked to digital platforms [3,10,11]. Among these efforts, the Disinformation Governance Board in the United States [12], withdrawn after criticism of its potentially censorial character, and the Global Disinformation Index in the United Kingdom [10], accused of operating with opaque criteria and ideological bias, stand out as illustrative cases. These models have also exposed additional risks such as concentration of discursive power, lack of transparency, and possible violations of informational pluralism.

Within the European context, structures such as the European Digital Media Observatory [13] and the East StratCom Task Force [14] have attempted to establish shared verification standards. However, reports from the European Court of Auditors and several governance studies point out that normative responses remain uneven, with persistent limitations in coordination, traceability, and legitimacy of interventions [3,9,15,16].

On the other hand, technical initiatives have been developed to design structural, objective, and replicable metrics for analyzing the narrative behavior of informational sources. These include studies on the cognitive persistence of disinformation, propagation mechanisms in social media, sociopolitical effects of pseudo-media, and the applications of big data and natural language processing (NLP) for detecting falsehoods [9,17]. While these contributions have advanced the understanding of the phenomenon, recent literature emphasizes their limitations: lack of conceptual consensus, limited impact on journalistic routines, difficulty distinguishing between editorial stance and deliberate manipulation, and low transparency in verification processes [3,18,19,20].

In this context, a methodological alternative is needed, one oriented toward citizens’ informational autonomy, that goes beyond merely detecting falsehoods and enables the evaluation of the structural quality of information received by individuals. Such an approach should identify the narrative frameworks through which public discourse is constructed. The goal is not to restrict interpretive freedom, but rather to strengthen critical capacity and foster conscious decision-making in an environment saturated with divergent narratives.

This study proposes a new paradigm of entropic structural analysis, which conceives disinformation not as a factual deviation but as a problem of narrative disorder and loss of epistemic coherence within open informational systems. The model is articulated through the concept of informational utility (UI), understood as an estimation of the epistemic value that a set of narratives provides for interpreting an event and constructing a coherent understanding. This utility is operationalized through the QVP-RI (Relational Information Valuation) operator. This dynamic metric combines informational entropy and normalized structural divergence to quantify the degree of disorder among sources without issuing truth judgments.

The proposed model is developed within the epistemic framework of open and democratic systems, in which the plurality of sources constitutes a structural condition of validity. Historically, the field has evolved from verificationist approaches, which focus on checking the correspondence of isolated facts to reality, to narrative approaches, which analyze the framing and semantic structures of discourse. We propose a third step: a structural–entropic approach. This lineage draws not only on information theory but on the systemic philosophy of Floridi [21] and the semiotics of Lotman [22], positing that disinformation is best understood as a measure of entropy, taken as the degree of disorder and incoherence within the ecosystem itself, rather than merely a collection of false statements.

This stance directly responds to Bertrand Russell’s [23] objection that a system of beliefs may be perfectly coherent yet entirely false if it lacks plural contrast. The same issue is historically evident in the persistence of the Black Legend, which operated within a closed communicative environment, where a single narrative matrix succeeded in consolidating a lasting and distorted perception of the Spanish Monarchy. Consequently, the model applies exclusively to informational environments where multiple narratives coexist and can be compared, ensuring that the measured coherence results from epistemic diversity rather than discursive closure.

This work offers a threefold contribution: theoretical, by redefining the notion of informational utility from the standpoint of applied epistemology; methodological, by operationalizing this approach through the QVP-RI index; and empirical, by validating its ability to quantify structural coherence and narrative divergence in real communicative contexts. The model thus provides a complementary tool to enhance informational autonomy, improve the traceability of media ecosystems, and anticipate structural risks of disinformation.

Following this conceptual framework, the subsequent sections develop the model’s computational formalization, empirical validation, and practical application: Section 2 presents the theoretical and epistemological foundations of the proposed approach; Section 3 details the computational formalization of the QVP-RI index, its calculation procedure, and empirical validation; Section 4 illustrates a practical application of the model within a real informational context; Section 5 reports, interprets, and discusses the results, as well as the implications of the model for disinformation research and the governance of media ecosystems.

## 2. Theoretical and Conceptual Framework

The objective of this section is to theoretically situate the hypothesis guiding this study: that narrative divergence constitutes a structural manifestation of disinformation, and that its measurement allows for the representation of the informational order within a communicative ecosystem.

From this premise, the study aims to demonstrate that disinformation can be described and quantified not by the falsity of its content, but by the degree of semantic disorganization separating the narratives produced about the same event within a plural and democratic epistemic framework.

On this basis, the following sections establish the conceptual and operational foundations of the proposed model, which interprets disinformation as a phenomenon of structural divergence among narratives and introduces a quantitative, replicable metric capable of representing it with analytical precision.

### 2.1. Citizen Autonomy and Access to Information

Citizen autonomy depends on the quality and coherence of the informational environment in which judgments are formed. When that environment becomes fragmented or distorted, the ability to decide freely is compromised, since the understanding of facts comes to rely on partial, contradictory, or biased narratives [24,25].

As Harari [26] points out, human societies are sustained by shared narratives that give meaning to reality and make cooperation possible. When those narratives are manipulated or diverge from one another, the common framework of meaning that enables public deliberation and collective action is eroded.

Disinformation primarily arises from the instrumentalization of a narrative by political, economic, or identity-based actors seeking to shape perceptions and behaviors [21]. In this process, the digital environment does not create disinformation but amplifies its effects, accelerating dissemination, segmenting audiences, and prolonging the persistence of content [27,28].

From this perspective, understanding disinformation requires analyzing how information is organized narratively and how the divergence between these discursive structures may influence the individual and collective perception of facts.

The citizens’ informational autonomy depends not only on access to data but also on the ability to recognize the coherence or narrative divergence of the symbolic ecosystem in which their judgment is formed [21].

### 2.2. Facts and Narratives: From Reference to Interpretation

Facts constitute the empirical foundation upon which narratives are built. Since Aristotle, truth has been understood as the correspondence between thought and reality (adequatio intellectus et rei). Kant reformulated this notion by showing that knowledge is not a passive reflection of objects but an active synthesis between experience and the categories of understanding. Facts, understood here in a broad sense as verifiable events that serve as the empirical reference of discourse, do not appear directly: they are constructed through language, perception, and the conceptual frameworks that give them meaning [29,30].

In this sense, information is not a mirror of the world, but a system of representation mediated by signs and cultural conventions [31,32]. A fact is not a raw datum but a shared reference unit whose value depends on the stability of the context and the social trust placed in the sources that sustain it [33,34].

Narrative is the form through which facts acquire meaning. Bruner [35] conceives it as a narrative structure that organizes temporal experience and translates events into an intelligible sequence. Every narrative selects, hierarchizes, and frames facts within a system of values and communicative purposes [36,37].

In this process, narration does not merely inform but constructs coherence, legitimizes perspectives, and emotionally orients collective interpretation. As Nietzsche observed, there are no pure facts, only interpretations that express a will to meaning. From a pragmatic perspective, Habermas [24] reminds us that understanding is built through communicative interaction, and Lotman [22] shows that cultural systems delimit the possible frames of interpretation.

**Figure 1:**
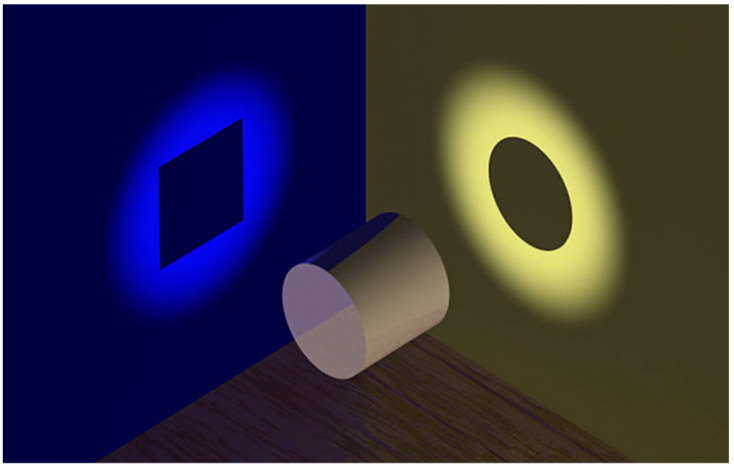
The same fact projects divergent representations depending on the angle of observation. This metaphor illustrates how the plurality of narratives generates narrative entropy and conditions the informational utility available to citizens.

Public narratives, by integrating or distorting facts, define the cognitive horizons through which societies make sense of the world [38]. Within this framework, the relationship between facts and narratives may be conceptualized through the metaphor of the cylinder (Figure 1): the same object generates different projections depending on the viewing angle. Each projection captures a partial aspect of reality, yet none provides an exhaustive representation.

The phenomenon of narrative divergence emerges when these projections cease to be recognized as complementary and are presented as mutually exclusive. At that point, the legitimate plurality of perspectives turns into fragmentation of the narrative order, weakening the structural coherence of the informational system.

Furthermore, informational divergence not only alters this structural coherence but also affects the emotional dimension of citizens. Recent research has shown that prolonged exposure to contradictory or incoherent messages can lead to confusion, fear, and distrust, thereby impairing decision-making capacity and ultimately affecting both individual and collective autonomy [38].

Between fact and narrative lies a space of mediation in which the very possibility of shared knowledge is decided. This mediation, far from being a defect of language, constitutes the structural condition of possibility for any communicable truth.

Recent studies in information science confirm that the structural coherence of communicative environments is positively correlated with epistemic trust and the perception of credibility among users [39,40].

A critical distinction must be made between democratic pluralism and disinformation. In a healthy pluralistic system, divergent narratives act as complementary projections (the square and the circle) that allow the citizen to mentally reconstruct the cylinder. This state maintains a manageable level of entropy where integration is possible. Structural disinformation arises when divergence becomes absolute, when the “square” narrative actively negates the existence of the “circle,” destroying the semantic links required to perceive the cylinder. Our model quantifies this state of disintegrative atomization. An entropy value approaching 1.0 does not signal a rich diversity of views, but a total failure of the ecosystem to sustain a shared reality, rendering the informational utility (UI) of the system null.

Narrative divergence should not be confused with disinformation. The former describes the degree of semantic dispersion among narratives addressing the same event; the latter arises when that divergence is intentionally induced or amplified to steer public perception. Recognizing this difference prevents misinformation from being reduced to isolated failures and allows it to be analyzed as a structural effect derived from the way narratives are organized and compete.

These theoretical foundations also provide the conceptual basis for a quantitative representation of narrative divergence.

### 2.3. Truth as Perspective

The concept of truth has undergone continuous revision throughout the history of thought. In classical philosophy, Plato defined truth as correspondence with the intelligible world, while Aristotle linked it to the correspondence between intellect and reality (adequatio intellectus et rei). Saint Augustine transposed this idea to the interior realm, conceiving truth as rational illumination derived from the logos of the divine. Kant transformed this paradigm by situating knowledge within the interaction between subject and object: truth ceased to reside in the object. It came to depend on the structural conditions of human understanding. In the twentieth century, Heidegger reinterpreted truth as aletheia, a process of unveiling through which Being partially reveals itself to human comprehension, and Gadamer [41,42] consolidated this hermeneutic view, understanding truth as the outcome of an interpretive dialogue between historical horizons.

In this transition toward relational conceptions of truth, Bertrand Russell’s [23] objection becomes pivotal: a system of beliefs may be perfectly coherent within itself and yet entirely false if it lacks contrast with other systems. This limitation of coherencies, the possibility of fictitious coherence, highlights the necessity of a plural epistemic framework, in which truth does not arise from the internal consistency of a single narrative, but from the structural comparison among coexisting narratives. In this sense, informational plurality is not a normative value but a structural condition for the mutual verification of discourses and, consequently, for the very possibility of shared knowledge. The proposed model aligns with this perspective: by analyzing narrative divergence within open ecosystems, it seeks to represent the epistemic quality of information not through the isolated truth of a single discourse, but through the ordered interaction of multiple narratives that coexist in the public sphere.

This evolution reflects the transition from an absolute conception of truth toward a relational and contextual one, dependent on the cultural frameworks and languages through which knowledge is expressed. In the field of contemporary communication, this shift acquires practical relevance: truth is no longer perceived as a stable entity, but rather as an emergent property of coherence among multiple representations. Arendt [43] warns that political truth relies on the existence of verifiable facts, yet its recognition depends on the collective will to preserve them. Foucault [44] expands this notion by showing that every regime of truth is structured by power relations that determine the legitimacy of discourse, while Habermas [24] proposes a deliberative conception in which truth emerges from communicative processes oriented toward mutual understanding. Lyotard [45] situates this debate within postmodernity, describing the crisis of legitimation that occurs when grand narratives lose authority in the face of a plurality of fragmentary narratives.

The tension between fact, interpretation, and power is exemplified in the dialogue between Jesus and Pilate (Jn 18:38) [46]. Pilate does not question the empirical event itself but the very possibility of a recognizable truth. This scene illustrates the distance between fact and narrative: while the former retains its existence, the latter contests its meaning. In epistemological terms, it represents the fracture between the ontological dimension of truth and its narrative dimension, a rupture that, in today’s digital ecosystem, is amplified on a global scale.

In contemporary informational environments, characterized by speed, hyperabundance, and algorithmic personalization, truth can no longer be understood as static correspondence but as structural coherence within an open and plural narrative system [21,26]. The epistemological question thus shifts from isolated verification to the relational evaluation of informational order. Determining the degree of truth within a communicative environment, in this framework, is equivalent to assessing the level of consistency and narrative convergence that sustains the intelligibility of the public sphere.

Conceiving truth as an emergent property of discursive systems allows disinformation to be analyzed not as a simple opposition between truth and falsehood, but as a disturbance of the structural balance that underpins collective understanding. Narrative divergence, in this sense, can be interpreted as an indicator of informational entropy, reflecting the semantic distance among versions of the same event. When strategic interests or polarization dynamics amplify such divergence, the system loses epistemic coherence, and its informational utility for citizens diminishes.

This relational framework is, in practice, the starting point from which the model we propose is articulated. The following section presents the operational formulation of the model, which translates these principles into a quantitative representation of the degree of narrative coherence and dispersion, enabling the estimation of the informational structure of a set of narratives and their capacity to sustain shared knowledge [21,47]. This approach establishes a methodological bridge between the theory of knowledge and the empirical analysis of information, translating epistemological principles into reproducible and verifiable metrics.

Building on these theoretical foundations, the following section introduces the operational formalization of the QVP-RI model, which translates the previously outlined epistemological principles, coherence, divergence, and informational order, into a quantitative representation of the degree of narrative disorganization within a communicative system. This formulation enables a transition from conceptual analysis to reproducible computation, preserving the connection between epistemology and information theory.

## 3. Formalization of the Model for Measuring Disinformation

This section presents the formalization of the model for measuring structural disinformation, understood as narrative divergence and loss of epistemic coherence among sources. The model is operationalized through the QVP-RI index, which combines informational entropy and structural divergence to estimate the narrative disorder of the system. Complementarily, UI is defined as an indicator of the epistemic value of a set of narratives derived from their structural organization.

The following subsections present the quantitative formulation of the index, including the entropic functions employed, the calculation procedure, and the discretization criteria. The definition and estimation of UI are specified, and the empirical validation procedure of the model and operator is described within real communicative contexts.

### 3.1. Quantitative Formalization

Let D=d1,d2,…,dn denote the set of discursive dimensions that describe different aspects of discourse, structural, semantic, emotional, or rhetorical, treated as independent from one another. All dimensions refer to the same event but capture distinct forms of narrative representation.

Each narrative unit (e.g., a headline or informational fragment) is represented by a feature vector [p, i], where p denotes semantic polarity and i represents intensity or expressive strength. These values are derived from the lexical and syntactic analysis of the text and normalized within the continuous range [−1, 1], ensuring comparability across heterogeneous dimensions.

Each dimension dᵢ is normalized to the range [0, 1] using a min–max linear adjustment [48], allowing heterogeneous metrics to be integrated into a shared analytical space.

To estimate the probability distribution of each dimension, continuous values are transformed through equidistant binning with k intervals, a standard procedure in entropic estimation for continuous data [49,50]. The optimal number of intervals is determined by an entropic convergence criterion, which identifies the point at which additional resolution no longer contributes significant information (∆H/∆H ≈ 0). In the experimental tests, equilibrium was consistently reached around k = 7, ensuring sufficient informational segmentation without overfitting and maintaining comparability across studies [49,50]. This parameter can be adjusted according to the sample size or analytical precision, while maintaining coherence across experiments.

To quantify narrative coherence loss, the model employs Shannon entropy [47] as the reference measure to estimate the informational variability of narratives. The choice of this measure stems from its universal applicability and its ability to describe the degree of disorder across systems with differing distributions.

Shannon’s formulation represents the most general expression of informational dispersion satisfying the axioms of continuity, maximality, and additivity [50], ensuring comparability across heterogeneous dimensions and the formal consistency of the index.

Nevertheless, the framework remains open to alternative entropic or divergence-based formulations, such as those of Rényi [51], Tsallis [52], Kullback–Leibler [53], or Jensen–Shannon [54], provided they preserve the model’s formal coherence and normalization within the range of [0, 1]. In this sense, the Shannon-based version should be regarded as a canonical implementation within an extensible paradigm for measuring narrative disorder, fully compatible with future adaptations and empirical comparisons [51,52,53].

The normalized Shannon entropy for each dimension is defined as follows:

(1) $$H_{i}=-\frac{1}{\ln\left(k\right)}\sum_{j=1}^{k}p_{ij}\cdot ln\left(p_{ij}\right),\;such\;that\;H_{i}\in [0,\;1]$$

where $p_{ij}$ represents the empirical probability of category j in dimension $d_{i}$, estimated as the relative frequency of values falling within each interval.

The value $H_{i}$ expresses the degree of informational dispersion within the dimension: values close to 0 indicate coherence (low variability), whereas values near 1 reflect disorder or semantic fragmentation.

It is well-documented that maximum likelihood estimators of Shannon entropy can underestimate true entropy when the sample size (N) is limited relative to the number of bins (k). To ensure statistical rigor and address potential small-sample bias, we apply the Miller–Madow correction to our estimator:

(2) $${\hat{H}}_{i}=H_{i}+\frac{k-1}{2N}$$

Each dimension contributes to the global index with an entropic weight $a_{i}$, reflecting its informational relevance and satisfying the following condition:

(3) $$\sum_{i=1}^{n}\alpha_{i}=1$$

In its basic formulation, the weights are assigned uniformly as $a_{i}=1/n$, to preserve analytical independence among dimensions. Alternatively, they may be derived from an independent calibration corpus, so that dimensions exhibiting greater informational variability acquire a higher relative weight. The objective is to prevent the same source of variability from being counted twice and to maintain the interpretative stability of the index.

The global narrative divergence index is defined as the weighted combination of the individual entropies:

(4) $${QVP}_{RI}=\sum_{i=1}^{n}\alpha_{i}\cdot H_{i},\;such\;that\;{QVP}_{RI}\in [0,\;1].$$

Low QVP-RI values indicate high narrative coherence (low disorder), whereas high values reflect semantic fragmentation or divergence among accounts.

The QVP-RI does not determine which account is more truthful but quantifies the degree of structural incoherence among narratives describing the same event. Therefore, it constitutes a relational indicator of disinformation, consistent with contemporary approaches that interpret it as a structural phenomenon of narrative divergence and loss of epistemic coherence [23].

The procedure can be implemented in any natural language analysis environment, ensuring its replicability and empirical applicability in studies of informational divergence.

### 3.2. Information Utility

Based on the value obtained from the QVP-RI index, UI is defined as a complementary measure that estimates the epistemic value of a set of narratives.

UI does not assess the factual accuracy of the content, but rather its structural coherence and ability to provide an ordered and intelligible informational environment in which the receiver can construct grounded and verifiable interpretations.

Information Utility is defined as the logical complement of the narrative divergence index:

(5) $$UI=1-{QVP}_{RI}$$

where $UI\in \left[0,1\right].$

High values indicate high narrative coherence and, therefore, greater epistemic utility; low values reflect discursive fragmentation and reduced cognitive usability. Both indices share the same mathematical structure and a common informational space of normalized discursive dimensions, ensuring their comparability.

- **Interpretation of UI**

UI reformulates semantic divergence in terms of cognitive usability, expressing the capacity of the narrative system to sustain understanding and shared knowledge. The more structured and coherent the collective narrative, the greater its utility for facilitating comprehension and informed decision-making.

An environment with low divergence fosters the construction of verifiable knowledge and reduces interpretative uncertainty, whereas a highly fragmented environment multiplies ambiguity and hinders the identification of shared facts.

- **Relation to Cognitive Autonomy**

From this perspective, UI can be interpreted as an indirect measure of cognitive autonomy, understood as the individual’s ability to analyze, evaluate, and contrast information without relying on external filters or centralized authorities.

Empirical evidence from media literacy research indicates that structured and coherent informational environments strengthen individuals’ capacity for critical evaluation and autonomous judgment. A meta-analysis of 51 media literacy interventions confirmed significant positive effects (d = 0.37) on media knowledge, critical understanding, perceived realism, and behavioral outcomes [55].

In a broader sense, this relationship can be framed within Floridi’s [21]. Epistemic model, according to which informational order constitutes the structural basis of reliable knowledge. From an empirical perspective, the findings of [38] demonstrate that the loss of narrative coherence not only reduces the intelligibility of the informational environment but also affects the receiver’s emotional stability, weakening their cognitive autonomy.

Thus, a sustained decrease in UI can be interpreted as a symptom of epistemic opacity, where information becomes less useful for autonomous reasoning and more vulnerable to polarization or manipulation.

- **Theoretical Behavior**

**Figure 2:**
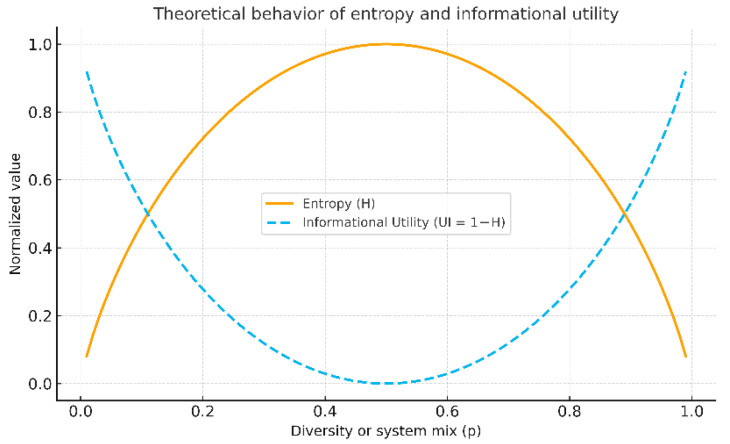
Theoretical behavior of entropy (H) and information utility (UI).

The theoretical behavior of entropy and its logical complement, UI, is illustrated in Figure 2. As narrative diversity increases, entropy grows rapidly until it reaches a saturation point representing the maximum possible informational disorder. Conversely, UI decreases progressively, reflecting the loss of epistemic coherence in the system.

This behavior confirms the complementary nature of both metrics and their internal consistency as indicators of informational order and cognitive autonomy associated with the comprehension process.

Entropy increases with narrative diversity and stabilizes when reaching maximum informational disorder. Information utility (UI = 1 − H) behaves inversely, indicating the degree of epistemic coherence available in the system.

Although the QVP-RI model is formulated for *n* discursive dimensions, the empirical validation presented below is performed on a single dimension. This decision is based on an experimental criterion: to isolate and verify the behavior of the index in its most elementary form before extending it to multidimensional configurations.

The following section describes the empirical validation procedure used to test the relationship between narrative divergence and information utility.

### 3.3. Empirical Validation

To empirically validate the sensitivity and consistency of the model, the calculation of the QVP-RI index was performed exclusively on the statistical properties of the texts. At the same time, the utility assessment tasks reflected the participants’ subjective perception. This distinction ensures that the experiment does not conflate the objective measurement of narrative divergence with the cognitive evaluation of comprehension, thereby allowing for the analysis of the correlation between structural disorder and perceived epistemic clarity.

The validation of the QVP-RI model was conducted through a controlled experiment designed and implemented by the Applied Social Research Center (CISA) at the University of Málaga to test its sensitivity to different degrees of narrative divergence and its stability in the presence of neutral content.

A total of 500 participants were recruited through the CISA’s online survey platform, stratified by gender, age, and educational level. The sample size ensured a statistical power greater than 0.80 to detect differences of at least 0.10 units in QVP-RI (α=0.05).

The experiment was conducted in a standardized digital environment, ensuring homogeneous exposure conditions and automatic recording of responses. Participants were randomly assigned to two experimental conditions:

- Experimental group, exposed to real media pieces presenting divergent narratives about the same geopolitical event, drawn from three leading digital sources.
- Control group, which received neutralized versions produced through factual intersection, retaining only the statements shared across the three verified sources.

To avoid redundancy, the neutralized versions were generated using a single factual intersection strategy, eliminating any interpretive or evaluative component. The semantic neutrality of the control material was verified through a mean polarity analysis (μ = 0.02) and inter-coder evaluation (α = 0.84), confirming the absence of relevant bias.

Semantic polarity was calculated using the VADER model [56], adapted to Spanish through lexical adjustment and contextual weighting of intensifiers and negations. Calibration was performed with a corpus of 10,000 journalistic headlines, achieving a mean correlation of r = 0.86 (*p* < 0.001) with human annotations. This adaptation, referred to as VADER-ES, preserves the methodological transparency and high reproducibility of the original model, outperforming transformer-based solutions that offer lower interpretability.

Two independent coders manually reviewed the results to ensure semantic equivalence between the neutralized and original pieces. Coherence and utility tasks exhibited adequate internal reliability (α = 0.86). Attention-check items were included, and 7% of inconsistent responses were excluded.

After exposure, participants completed tasks assessing summarization, factual coherence, and perceived utility, which enabled the computation of both the QVP-RI index and its complementary measure, UI. UI thus functions as an inverse measure of narrative disorder, allowing for empirical comparison between measured structural coherence and perceived epistemic clarity. UI is not calculated independently but is derived directly as the complement of the index (UI = 1 − QVP-RI). At the same time, its subjective evaluation acts as a convergent variable to empirically validate perceived coherence. Assumptions of normality and homogeneity of variance were verified and met (Kolmogorov–Smirnov, *p* > 0.05; Levene, *p* > 0.10).

Results showed a mean QVP-RI = 0.70 (95% CI: 0.66–0.74) in the experimental group, indicative of high semantic divergence and informational fragmentation, compared to QVP-RI = 0.18 (95% CI: 0.15–0.21) in the control group. The difference was statistically significant (t (498) = 18.42, *p* < 0.001).

Complementarily, the mean UI was 0.30 in the experimental group and 0.82 in the control group, revealing a clear difference in epistemic coherence between conditions. The index exhibited high internal consistency (α = 0.87) and computational stability above 95% in repeated-sampling (bootstrapping) tests, demonstrating that observed variations reflect genuine narrative differences rather than statistical noise. These reliability tests confirm that the QVP-RI is a robust and replicable structural measure, independent of the conceptual correlation between divergence and epistemic clarity.

Additionally, an exploratory comparison with conventional dispersion measures such as variance and the Gini coefficient showed high correlation without complete overlap, suggesting that QVP-RI captures structural components beyond mere statistical dispersion. In future extensions, a systematic sensitivity analysis is planned, incorporating alternative metrics such as Kullback–Leibler divergence and Rényi entropy, to assess the model’s relative performance and extend its comparative validation [53].

The experiment was designed under the assumption of a plural informational ecosystem, in which divergent sources represent legitimate perspectives on the same event. This framework allows the results to be interpreted as a measure of epistemic order within open systems, avoiding extrapolation to homogeneous or controlled communication contexts.

Overall, these findings confirm the convergent and discriminant validity of the QVP-RI model, demonstrating its ability to robustly detect semantic divergence between narratives. They also reinforce their practical utility in analyzing real informational environments, where narrative divergence can serve as an early indicator of disinformation or loss of communicative coherence in open ecosystems.

## 4. Application Example

To illustrate the applicability of the QVP-RI index, narrative divergence was analyzed in the media coverage of the Israel–Gaza conflict in September 2024, approximately one year after the 7 October 2023 attacks. This case is selected as a high-divergence benchmark to test the behavior of the QVP-RI index under conditions of extreme narrative polarization; comparable levels of entropic dispersion would be expected in any highly contested public event, independently of its specific geopolitical content.

The analysis was designed to answer the following question: How do Spanish media sources portray and narratively represent Israel in their coverage of the Gaza conflict one year after the attacks of 7 October 2023?

To address this question, the study measured the degree of narrative divergence among Spanish media by analyzing the polarity and intensity of their discursive framing of the same event, avoiding any political or normative evaluation.

In this case, the analysis focused on the dimension of narrative polarity, measured on a continuous scale [−1, 1]. Although the entropic computation is applied to a single variable, it simultaneously encodes two semantic components: the orientation of the discourse (the sign of the polarity) and the intensity of positioning (the absolute magnitude). Therefore, the model is implemented as unidimensional from an operational standpoint but retains a bidimensional interpretation in terms of narrative direction and intensity.

It should be noted that although these two components are conceptually distinct, they do not constitute independent dimensions in a formal sense, since both derive from the same semantic continuum. Intensity is a function of the absolute value of polarity, and therefore, a mathematical dependency exists between them. Consequently, the entropic calculation is performed on a single practical dimension, ensuring mathematical coherence and avoiding informational redundancy.

A corpus of 118 headlines was collected from nine primary Spanish digital sources (ABC, El Confidencial, El Español, El Mundo, El País, El Periódico, La Razón, Libertad Digital, and Okdiario).

The selection of the 118 headlines was designed as a controlled divergence benchmark aimed at testing the sensitivity and stability of the QVP-RI index under extreme informational conditions. The corpus was constructed within a delimited temporal window centered on the focal event (September 2024), incorporating media outlets with diverse editorial orientations in order to maximize narrative variability.

Explicit exclusion criteria were applied to reduce informational noise and structural redundancy. Duplicate headlines, substantially equivalent reformulations, and contents only tangentially related to the focal event were excluded from the sample. This procedure ensured that observed divergence reflected genuine narrative differences rather than artifacts of repetition or peripheral coverage.

Under these conditions, the corpus should not be interpreted as a statistically representative sample of the media ecosystem, but as a controlled structural test set, consistent with its role as a benchmark for evaluating narrative divergence and the robustness of the proposed index.

Each headline was processed through a narrative polarity analysis toward Israel, using a continuous scale [−1, 1], where −1 represents a negative orientation and +1 a positive orientation, applying the VADER method [56]. The resulting values were discretized into seven equidistant categories, in accordance with the operational procedure described in Section 3.

The resulting frequencies were transformed into empirical probabilities to compute the normalized Shannon entropy, which represents the QVP-RI value for the polarity dimension. Table 1 shows the mean, normalized, and interval polarity values for each information source.

From this distribution, a normalized entropy of H = 0.987 was obtained, corresponding to a QVP-RI index = 0.987 and a UI = 0.013. Given our case study parameters (N = 118, k = 7), the bias correction term is approximately 0.025. This confirms that the high entropy values observed are not stochastic artifacts but significant structural indicators.

These results reveal an extreme level of narrative divergence among the analyzed media outlets: although they report on the same events, their interpretive frameworks differ substantially.

From an informational perspective, the media ecosystem exhibits a near-maximum degree of narrative disorder and minimal epistemic utility, which severely limits citizens’ ability to construct shared and well-founded interpretations.

The observed entropy value (H = 0.987) constitutes a direct empirical manifestation of what we conceptualize as the mutual negation of narrative projections. In this configuration, different media sources produce internally coherent narrative representations of the same event that become structurally incompatible when projected onto a shared interpretive space.

This dynamic operationally reflects the logic of the ambiguous cylinder metaphor: each narrative projection is locally consistent, yet the system as a whole lacks a unified representational structure. An entropy value close to the theoretical maximum, therefore, indicates not merely informational dispersion, but a loss of epistemic order, where the coexistence of divergent narratives prevents the reconstruction of a common frame of intelligibility.

In this sense, the metaphor functions not only as an illustrative device but as a structural abstraction that becomes directly observable in empirical data through entropic measurement.

**Figure 3:**
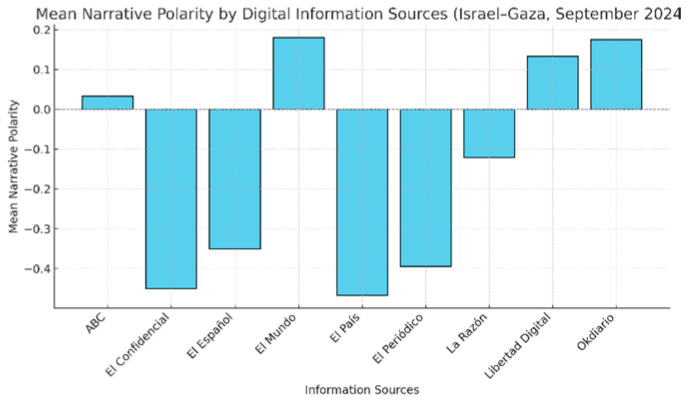
Narrative polarity dispersion among Spanish information sources on the Israel–Gaza conflict (September 2024).

As shown in Figure 3, the dispersion of narrative polarity across media outlets reveals substantial semantic divergence in their framing of the same events, visually illustrating the structural fragmentation measured by the QVP-RI index.

In operational terms, this case illustrates the QVP-RI’s ability to quantify narrative fragmentation and variation in discourse intensity within real-world contexts, providing a structural and traceable measure of epistemic coherence that does not rely on editorial judgments or truth-verification procedures. The extreme value observed reflects the high narrative polarization characteristic of certain international events and confirms the model’s sensitivity to scenarios of pronounced discursive dispersion.

This example, therefore, illustrates the applicability of the QVP-RI model in real informational analysis contexts, demonstrating its potential as a quantitative tool for structural evaluation.

## 5. Discussion and Conclusions

This study proposes an entropic structural model for analyzing disinformation, conceived as a phenomenon of narrative divergence and loss of epistemic coherence within open informational systems.

The model redefines the traditional approach to the problem by shifting the focus from factual verification toward the relational structure of public knowledge, where disinformation manifests as semantic disorder among narratives competing for the interpretation of shared events.

Although the present study is deliberately formulated as a unidimensional design, intended as a proof of concept for the feasibility of the structural–entropic paradigm, the QVP-RI operator is naturally extensible to a multidimensional setting. This extension does not involve a simple aggregation of additional variables, but rather the explicit definition of narrative dimensions that are both informationally relevant and sufficiently orthogonal to sentiment polarity, which acts here as the core divergence axis in high-intensity informational contexts.

Operationally, a candidate dimension should satisfy two criteria: (i) it must capture a distinguishable structural property of public discourse (such as thematic focus, epistemic modality, or communicative function), and (ii) it should contribute non-redundant information with respect to sentiment polarity, which can be assessed through correlation or informational dependency measures.

Multidimensional integration can be formalized through a weighted entropy formulation, where each dimension contributes according to a weight *w_i_*, reflecting its relative informational relevance. These weights may be empirically derived or defined on the basis of explicit theoretical criteria. Under this approach, the global informational entropy of the system is expressed as a normalized combination of partial entropies across dimensions, preserving interpretability and ensuring methodological reproducibility. Importantly, this multidimensional formulation is presented as a methodological extension of the operator, not as an empirical claim within the scope of the present study.

This formulation allows the QVP-RI operator to scale to more complex narrative environments without compromising its conceptual coherence or structural foundations while maintaining a clear separation between the validated unidimensional implementation presented here and future multidimensional extensions.

The entropy-based model was implemented through the QVP-RI (Relational Information Valuation) index, which combines informational entropy and normalized structural divergence to estimate the epistemic order of public discourse. In this version, the implementation relied on Shannon entropy [46]. However, the framework remains open to alternative formulations—such as Rényi, Tsallis, Kullback–Leibler, or Jensen–Shannon—provided that formal coherence and index normalization are preserved. Thus, the QVP-RI represents a canonical instance within an extensible paradigm for measuring narrative disorder and structural coherence.

Empirical validation was conducted through a controlled experiment with 500 participants, designed to test the model’s sensitivity to varying degrees of narrative divergence and its stability when exposed to neutral content. Results revealed an inverse correlation between narrative divergence and informational utility (UI), confirming that the structural disorder of narratives measurably affects perceived epistemic clarity. The index exhibited high internal consistency (α= 0.87) and computational stability above 95% across repeated sampling tests, demonstrating its robustness and replicability as a structural metric.

As an illustrative example, the application to media coverage of the Israel–Gaza conflict (September 2024) demonstrated the model’s viability in real-world contexts and its ability to represent narrative divergence quantitatively, without resorting to editorial judgments or truth verification procedures.

Accordingly, the Israel–Gaza example should be interpreted as an extreme validation scenario aimed at testing the sensitivity of the QVP-RI index, rather than as a substantive or normative assessment of the conflict itself.

It is also important to define the scope of the narrative units analyzed. While traditional analysis focuses on prolonged discourse, this study utilizes headlines as proxies for narrative frames. In the digital attention economy, headlines often function as autonomous ‘micro-narratives’ that contain the essential semantic orientation of the story. While we acknowledge the ‘clickbait’ tendency noted in digital journalism, the headline represents the primary point of contact and semantic framing for most users, justifying its use as a valid unit for measuring structural divergence in this context.

Finally, we must address the ecological validity of the model regarding user consumption. While the QVP-RI operator measures the structural availability of divergent narratives, it does not account for algorithmic filtering or echo chambers that isolate users from this plurality. Individuals are often exposed to a single narrative strand by virtue of social sorting. Therefore, a system may possess high informational utility theoretically yet fail to strengthen critical capacity if users are structurally prevented from accessing contrasting narratives. The QVP-RI should thus be viewed as a metric of the ecosystem’s potential for coherence, rather than a direct measure of user consumption.

Together, the model and its implementation offer a quantitative and replicable way to study how narrative coherence varies in open information systems. Its design allows for the integration of multiple discursive dimensions, opening the possibility of developing more complex relational metrics aimed at analyzing informational plurality, institutional reputation, or public trust.

From an epistemological standpoint, the model remains unaffected by Bertrand Russell’s [19] coherentist paradox, as it operates within open ecosystems in which narrative diversity functions as a structural condition of validity. Consequently, the proposed approach does not aim to determine the truth of content but to evaluate the intelligibility and epistemic order of plural informational systems.

In short, the study proposes a consistent and coherent method for assessing the structural quality of information based on the degree of divergence between accounts.

## Figures and Tables

**Table 1:** Mean narrative polarity by Spanish digital media information sources (Israel–Gaza, September 2024).

Sources	Mean Polarity	Normalized Value	Interval
**ABC**	0.033	0.517	4
**El Confidencial**	−0.450	0.275	2
**El Español**	−0.350	0.325	3
**El Mundo**	0.180	0.590	5
**El País**	−0.467	0.267	2
**El Periódico**	−0.394	0.303	3
**La Razón**	−0.121	0.439	4
**Libertad Digital**	0.133	0.567	4
**Okdiario**	0.175	0.588	5

**Note:** Interval assignment follows an equidistant discretization over the normalized range [0, 1] using a left-closed, right-open binning scheme [a,b). Under this rule, near-boundary values may fall into adjacent intervals despite being numerically close.

## Data Availability

The original contributions presented in this study are included in the article. The first data source consists of news articles published by publicly accessible online media outlets. These data were collected through automated web scraping techniques applied exclusively to open access content. All sources are publicly available and do not require registration or restricted access. No personal data were collected, processed, or stored. The data consist solely of published journalistic content and were used exclusively for academic research purposes. The second data source consists of survey responses collected specifically for this study. Surveys were administered using internal data collection systems developed and managed by the Center for Applied Social Research (CISA). Participation was voluntary, and no personal or sensitive data were collected. Responses were anonymized at the time of collection and stored and analyzed exclusively in aggregated form. The survey followed a stratified sampling design based on key sociodemographic variables (gender, age group, and educational level) to ensure adequate representativeness of the study population, as described in the manuscript. The final sample consisted of 500 participants, providing sufficient robustness for the comparative analyses reported. Participants were informed about the purpose of the study, the voluntary nature of their participation, and the anonymous treatment of their responses. An informed consent statement was provided prior to participation.
